# Focused review on artificial intelligence for disease detection in infants

**DOI:** 10.3389/fdgth.2024.1459640

**Published:** 2024-11-25

**Authors:** Katrin D. Bartl-Pokorny, Claudia Zitta, Markus Beirit, Gunter Vogrinec, Björn W. Schuller, Florian B. Pokorny

**Affiliations:** ^1^ Division of Phoniatrics, Medical University of Graz, Graz, Austria; ^2^ EIHW – Chair of Embedded Intelligence for Health Care and Wellbeing, University of Augsburg, Augsburg, Germany; ^3^ CHI – Chair of Health Informatics, Technical University of Munich, Munich, Germany; ^4^ Center for Interdisciplinary Health Research, University of Augsburg, Augsburg, Germany; ^5^ Munich Center for Machine Learning (MCML), Munich, Germany; ^6^ GLAM – Group on Language, Audio & Music, Imperial College London, London, United Kingdom

**Keywords:** artificial intelligence, machine learning, deep learning, infancy, disease, detection, prediction, healthcare

## Abstract

Over the last years, studies using artificial intelligence (AI) for the detection and prediction of diseases have increased and also concentrated more and more on vulnerable groups of individuals, such as infants. The release of ChatGPT demonstrated the potential of large language models (LLMs) and heralded a new era of AI with manifold application possibilities. However, the impact of this new technology on medical research cannot be fully estimated yet. In this work, we therefore aimed to summarise the most recent pre-ChatGPT developments in the field of automated detection and prediction of diseases and disease status in infants, i.e., within the first 12 months of life. For this, we systematically searched the scientific databases PubMed and IEEE Xplore for original articles published within the last five years preceding the release of ChatGPT (2018–2022). The search revealed 927 articles; a final number of 154 articles was included for review. First of all, we examined research activity over time. Then, we analysed the articles from 2022 for medical conditions, data types, tasks, AI approaches, and reported model performance. A clear trend of increasing research activity over time could be observed. The most recently published articles focused on medical conditions of twelve different ICD-11 categories; “certain conditions originating in the perinatal period” was the most frequently addressed disease category. AI models were trained with a variety of data types, among which clinical and demographic information and laboratory data were most frequently exploited. The most frequently performed tasks aimed to detect present diseases, followed by the prediction of diseases and disease status at a later point in development. Deep neural networks turned out as the most popular AI approach, even though traditional methods, such as random forests and support vector machines, still play a role—presumably due to their explainability or better suitability when the amount of data is limited. Finally, the reported performances in many of the reviewed articles suggest that AI has the potential to assist in diagnostic procedures for infants in the near future. LLMs will boost developments in this field in the upcoming years.

## 1 Introduction

Artificial intelligence (AI) can help humans to save time and resources by solving complex tasks with machine learning (ML) representing the underlying methodology of extracting knowledge from data and subsequently generalising to new, unseen data. Due to recent advancements including the rise of deep learning methodology—a branch of ML, AI has been gaining momentum in all domains of our lives. In medicine, current AI technologies focus on the detection of diseases as well as on monitoring of disease progression and intervention success (e.g., 1–3). AI systems cannot replace healthcare professionals, but they are intended to assist them in their decision-making process similar to other medical devices and diagnostic tools doctors regularly use. For optimal functionality of AI systems, huge amounts of data are needed, such as imaging data, audio recordings, and vital parameters (4–6). These data can either be collected in standardised conditions in laboratories or, alternatively, by means of wearable devices in the wild, i.e., in patients’ everyday environments (7, 8). The latter approach has certain advantages such as minimising the white-coat effect (9) or the possibility to collect long-term data. These can be used to capture symptoms that only occur sporadically over the day or to investigate the course of a disease over weeks, months, or even years (10, 11).

AI has been achieving promising performance especially in tasks related to the medical conditions cancer, mental diseases, and chronic diseases (12–14). In the last few years, healthcare-targeted AI research has also been focusing more and more on children and even infants, i.e., children in their first year of life – an especially vulnerable period. Timely diagnosis is especially important at this young age as it enables early intervention and therewith facilitates the best possible outcomes for affected individuals (15).

The first studies using AI systems for the detection of clinical signs in infants were carried out several decades ago (e.g., 16, 17). At the end of 2022, the release of ChatGPT—a sophisticated generative AI chatbot based on large language models (LLMs) which can process and generate natural language, heralded a new era of AI with serious implications for diverse sectors of life, including research and education, and with numerous application opportunities, amongst others in medicine (18–20). While the actual impact of LLMs remains to be estimated within the next couple of years, this review aims to shed light on the most recent pre-ChatGPT research activities in the field of automatic (ML-based) disease (status) detection^1^ and prediction in infants, which is why we decided to include only studies published from 2018 until 2022. Although there are reviews on AI applications in newborns (21) and on AI used for development monitoring of children up to 18 years (22), to the best of our knowledge, this is the first review of the recent pre-ChatGPT literature on automatic detection and prediction of disease (status) in the first year of life. We specifically aimed to identify and discuss investigated medical conditions, exploited data types, performed tasks, applied ML approaches, as well as achieved detection/prediction performances prior to the advent of LLMs.

## 2 Methods

A systematic literature review was done to capture developments between 2018 and 2022 in automatic detection and prediction of diseases and disease status in the first year of life. We performed the following steps: (1) specification of research questions, (2) decision on search engines, (3) definition of inclusion and exclusion criteria, (4) determination of search terms, (5) extraction of data, (6) identification of relevant articles, (7) review of selected articles for extraction of key findings.

### 2.1 Research questions

We decided to address the following research questions (RQs):

**RQ1:** How did the number of articles on automatic disease (status) detection and prediction in infants develop over time?
**RQ2:** Which medical conditions in infants were investigated by means of ML approaches?
**RQ3:** Which data were used for automatic disease (status) detection and prediction in infants?
**RQ4:** How is the distribution of tasks on detection and prediction of diseases and disease status?
**RQ5:** Which ML approaches were applied for automatic disease (status) detection and prediction in infants?
**RQ6:** Which measures were used to report the performance of automatic disease (status) detection and prediction in infants and how did ML approaches actually perform?

RQ1 refers to articles published between 2018 and 2022. As artificial intelligence is a very rapidly changing field, we decided to refer to articles published in 2022 only when answering RQ2–RQ6. This allowed us to capture the most recent trends preceding the release of ChatGPT.

### 2.2 Search strategy

In order to cover articles both from the technical and the medical community, we chose the search engines PubMed and IEEE Xplore. We defined the following inclusion and exclusion criteria: All articles needed to be original articles written in English and published in journals or conference proceedings in the years 2018 to 2022. Articles needed to focus on conditions that can be classified according to standard disease classification systems such as the International Classification of Diseases 11th Revision (ICD-11). Studies needed to have investigated infants, i.e., children in their first year of life; studies having focused on a broader age range were included if the authors explicitly stated that also individuals in their first year of life were investigated. Studies needed to have applied an ML approach to automatically detect, predict, or characterise a medical condition. In order to analyse whether data from the first year of life is useful for the detection or prediction of disease (status), at least parts of the data fed into the ML model shall have been collected during the participants’ first 12 months of life. Studies having only used data of the infants’ mothers or data collected prior to birth were excluded. Preterm birth without further complications in the neonatal period or later in childhood was not regarded as a condition of interest and related studies were excluded. Moreover, articles were excluded if the age or the ML methodology was not mentioned.

We applied the following search string to identify potentially relevant articles in PubMed and IEEE Xplore: “(artificial-intelligence OR machine-learning* OR deep-learning* OR neural-net*) AND (infant OR infan*) AND (disorder* OR disease* OR disabil* OR patho*).” In PubMed, the search was automatically limited to the first two years of life. The search revealed a total of 927 potentially relevant articles: 743 articles in PubMed and 184 articles in IEEE Xplore (last update of the literature search: 14 August 2023). All articles were evaluated for inclusion based on title and abstract by the first author (KDB-P) and by at least one of the authors MB, CZ, GV, or FBP. Whenever a rater was not able to determine whether an article fulfilled the inclusion criteria based on title and abstract, he or she read the full text to come to a decision. The full text of an article was also read in case of disagreement between the raters. Articles with disagreement were discussed within the team until consensus was achieved. This procedure resulted in a final number of 154 included articles: 125 from PubMed and 29 from IEEE Xplore. 129 were peer-reviewed journal articles and 25 were conference proceedings. A list of included articles is provided in a .bib-file in the Supplementary Material.

### 2.3 Extraction of key findings

In order to address RQ1, we extracted the publication year of each included article. In order to address RQ2, RQ3, RQ4, RQ5, and RQ6, we checked the 46 articles published in 2022 against the following details: RQ2) medical condition, RQ3) data used for automatic disease (status) detection or prediction, RQ4) performed tasks, RQ5) ML approach(es) used, and RQ6) achieved detection/prediction performance. Data extraction was performed based on the full text of the articles by KDB-P, CZ, and GV. FBP checked the extracted data for appropriateness and completeness. Entries with disagreement were discussed within the team until consensus was achieved.

## 3 Results

In the following, we present our research question-specific findings in separate subsections. Comprehensive information on the 46 reviewed articles from 2022 is given in Table S1 in the Supplementary Material. In order to answer RQ2–RQ6, we summarised details from this Table.

### 3.1 RQ1: publication trend

**Figure 1:**
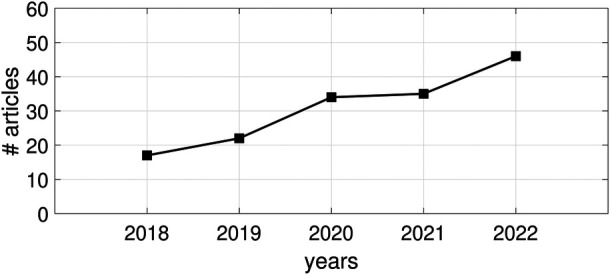
Publication trend in the field of automatic disease (status) detection and prediction in infancy in terms of number of (#) published articles between 2018 and 2022.

Within the investigated 5-years time window, a positive research trend can be observed; see Figure 1. Increasing research activity in the field of automatic disease status detection in infancy is reflected by a steadily increasing number of published articles starting with 17 articles in 2018 and ending up with 46 articles in 2022 except for a single slight stagnation in between in 2021.

### 3.2 RQ2: medical conditions

A total of 27 medical conditions were investigated in the framework of the 46 included articles published in 2022. We assigned the medical conditions to the respective ICD-11 categories (23). In case a medical condition—as stated in a given article—is not listed in the ICD-11, we assigned it to the best matching ICD-11 category. This was the case for example for the condition “Cognitive deficits” (24–26): ICD-11 (23) suggests to classify disorders characterised by developmental deficits in cognitive function to the grouping neurodevelopmental disorders. As “Neurodevelopmental disorders” belong to the category “Mental, behavioural or neurodevelopmental disorders,” we selected this ICD-11 category for the medical condition “Cognitive deficits.” Following this procedure, the included articles cover twelve ICD-11 categories. The category “Certain conditions originating in the perinatal period” was addressed most frequently, namely in twelve articles. It was also the category covering the highest number of different medical conditions, namely six. Details on the respective disease categories and medical conditions dealt with are provided in Table 1. Some of the articles dealing with the same medical condition have partly overlapping authors. This is the case for bronchopulmonary dysplasia (27–30), cognitive deficits (24, 25, 31), retinopathy of prematurity (32–34), and postnatal growth failure (35, 36).

**Table 1:** ICD-11 disease categories and related medical conditions dealt with in the 46 reviewed articles from 2022 with the respective number of articles in brackets. ICD-11 disease categories and medical conditions are listed (i) in decreasing order according to the number of articles assigned to the respective categories/conditions, and (ii) alphabetically.

ICD-11 disease category	Medical condition
Certain conditions originating in the perinatal period (12)	Bronchopulmonary dysplasia (4)
	Necrotising enterocolitis (4)
	Hypoxic-ischemic encephalopathy (1)
	Neonatal opioid withdrawal syndrome (1)
	Parenteral nutrition-associated cholestasis (1)
	Postnatal intestinal perforation (1)
Mental, behavioural or neurodevelopmental disorders (7)	Cognitive deficits (3)
	Autism spectrum disorder (2)
	Developmental dyslexia (1)
	Language deficits (1)
Developmental anomalies (6)	Congenital heart disease (2)
	Craniosynostosis (2)
	Coarctation of aorta (1)
	Developmental dysplasia of hip (1)
Diseases of the nervous system (5)	Cerebral palsy (2)
	Neonatal intraventricular hemorrhage (1)
	Neuromotor disorders (1)
	WEST epilepsy syndrome (1)
Diseases of the visual system (5)	Retinopathy of prematurity (5)
Certain infectious or parasitic diseases (4)	Sepsis (3)
	Serious bacterial infection (1)
Symptoms, signs or clinical findings, not elsewhere classified (2)	Postnatal growth failure (2)
Diseases of the ear or mastoid process (1)	Hearing loss (1)
Diseases of the respiratory system (1)	Neonatal chronic lung disease (1)
Diseases of the skin (1)	Atopic dermatitis (1)
Endocrine, nutritional, or metabolic diseases (1)	Diabetes mellitus type 1 (1)
Sleep-wake disorders (1)	Sleep apnea (1)

### 3.3 RQ3: data

**Figure 2:**
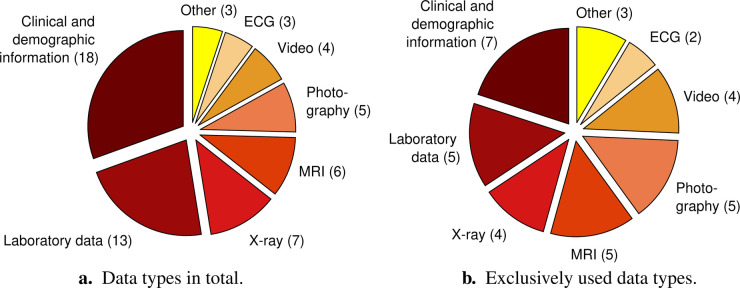
Distribution of data types dealt with in the 46 reviewed articles with the respective number of articles in brackets. The pie chart on the left **(a)** considers the total number of data types, i.e., it also includes multiple different data types used in one and the same study. The pie chart on the right **(b)** only comprises studies that exclusively exploited one single data type. The category “Other” summarises data types that only occur in one article, respectively. These are “Audio,” “Electroencephalography,” and “Thermal data” both for **(a)** and **(b)**. ECG, electrocardiography; MRI, magnetic resonance imaging.

Various different data types were used to automatically detect and characterise diseases in infancy. We classified the data in ten categories: clinical and demographic information, laboratory data, X-ray, magnetic resonance imaging (MRI), photography, video, electrocardiography (ECG), audio, electroencephalography (EEG), and thermal data. The category “clinical and demographic data” includes clinical details from pregnancy and birth, results of standardised assessments, information on treatment, comorbidities, and familial history, environmental data, socio-economic data, etc.; occasionally single laboratory parameters may be included, e.g., in case the authors did not specify the exact data acquisition approach for single features (e.g., whether oxygen saturation was collected via laboratory blood analysis or via pulse oximetry). The category “laboratory data” includes analysed blood and stool samples. Figure 2 presents the number of articles that used the respective data types for automatic disease status detection. The most widely used data type was clinical and demographic information, followed by laboratory data. Thirty-five articles relied on a single data type (see Figure 2b), nine articles (29, 37–44) on two data types, and two articles (45, 46) on three data types.

### 3.4 RQ4: tasks

To answer RQ4, each task was assigned to one or more of the following categories: “detection of condition” (demographic variables/measurements are used to detect a condition), “detection of condition status” (demographic variables/measurements are used to detect the status of a condition such as severity or subtype of condition), “prediction of condition” (analysis of demographic variables/measurements suggests how likely an individual is to develop a condition), “prediction of condition status” (demographic variables/measurements are used to predict long term outcome such as severity of a condition in childhood). A total of 51 tasks were performed by the 46 reviewed studies from 2022. Forty-eight of the 51 tasks were assigned to only one of the four predefined task categories. The greatest proportion of them, namely 17/48, aimed to “detect a condition.” Twelve tasks, respectively, aimed to “predict a condition” or “predict a condition status.” Seven tasks were assigned to the category “detection of condition status.” The remaining three tasks were each assigned to two categories, namely “detection of condition” and “detection of condition status.” Detection tasks included both tasks using data acquired in infancy (e.g., retinal images) and tasks using retrospectively acquired data (e.g., details on pregnancy) to detect a present condition or condition status.

### 3.5 RQ5: machine learning approaches

**Figure 3:**
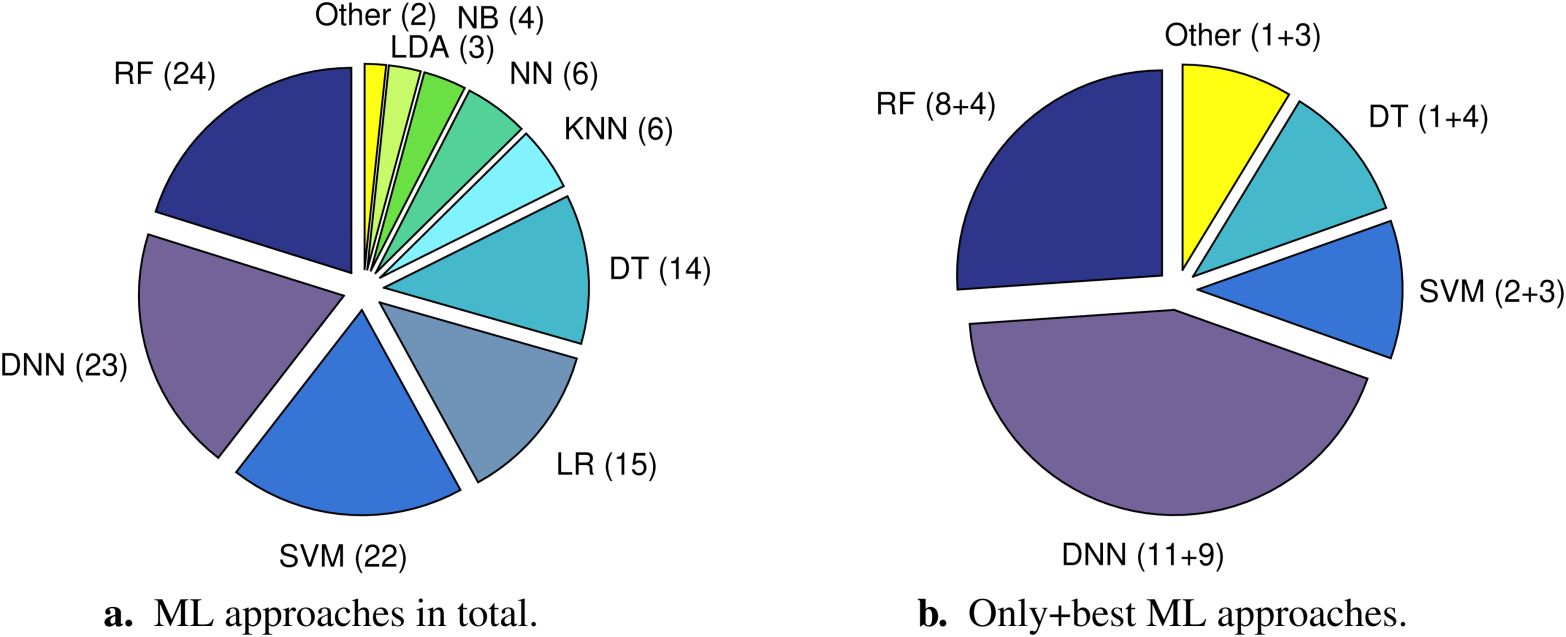
Distribution of machine learning (ML) approaches utilised in the 46 reviewed studies with the respective number of studies (articles) in brackets. The pie chart on the left **(a)** considers the total number of approaches, i.e., it also includes multiple different approaches tried out in one and the same study. The pie chart on the right **(b)** only comprises one approach per article, namely either the only approach that was used (left summand in brackets) or the respective best performing approach (right summand in brackets). The category “Other” summarises approaches that only occur in one article, respectively. These are “Decision tree + neural network (ensemble learning)” and “Hidden Markov model” for **(a)** and “Naïve Bayes,” “Decision tree + neural network (ensemble learning),” “Logistic regression,” and “Neural network” for **(b)**. DNN, deep neural network (in case a DNN with two hidden layers and a DNN with more than two hidden layers were tried out in the same study, DNN was just counted once); DT, decision tree; KNN, k-nearest neighbors; LDA, linear discriminant analysis; LR, logistic regression; NB, naïve Bayes; NN, neural network; RF, random forest; SVM, support vector machine.

Random forests, deep neural networks (DNNs), and support vector machines were most frequently used in the 46 reviewed studies from 2022; see Figure 3a. In exactly half of the studies, more than one ML approach was tried out. When only taking into account the respective best performing approach (as reported by the authors and/or evident from the given results, i.e., best results across the reported performance measures, see Subsection 3.6) of these studies plus the respective only approach of the rest of the studies, DNNs clearly come off as the top approach; see Figure 3b. In eleven studies DNNs were applied as the only approach, whereas in nine studies DNNs outperformed other approaches. In 11 out of the 14 remaining studies, in which ML approaches other than DNNs yielded the best performance, DNNs were not among the compared approaches. Thus, we only registered three studies, in which a non-DNN approach outperformed a DNN approach (31, 35, 37).

Please note that in case of different ML algorithms used in combination, e.g., one for representation learning (i. e., the use of ML to automatically discover the optimal data representation) and one for subsequent disease (status) detection/prediction, we only considered the ultimate approach. Moreover, we categorised ML approaches according to their underlying basic principle, irrespective of potential implementational specialties. For example, extreme gradient boosting (XGBoost) was assigned to the ML category “Decision tree.” Furthermore, we considered a neural network as deep if it was implemented with two or more hidden layers (i.e., more than three layers in total). In doing so, we additionally differentiated between DNNs with exactly two hidden layers and DNNs with more than two hidden layers; see Table S1 in the Supplementary Material. In case no information about the number of layers of a neural network is revealed in an article and the authors did not explicitly state that a deep architecture was used, we categorised the respective model as neural network only.

Cho et al. (37) employed an artificial neural network with exactly two hidden layers; (26) implemented a long short-term memory (LSTM) neural network with bi-directional structure (also two hidden layers). Han et al. (35) tried out different network architectures with the number of hidden layers varying from one (traditional neural network) to five (DNN with two and DNNs with more than two hidden layers). In all (20) other studies in which DNNs were applied, the respective networks were exclusively implemented with more than two hidden layers. Five studies explicitly disclose the use of established very deep network types, such as residual neural networks (ResNets) (29, 34, 47, 48), or a very deep convolutional neural network (VGG) (30).

### 3.6 RQ6: performance

Forty-four of the 46 reviewed studies from 2022 dealt with (binary) classification models, i.e., generating a discrete AI system output (e.g., 29, 47, 49, 50). Just four studies additionally or exclusively investigated regression models, i.e., generating a continuous AI system output (32, 33, 51). Across the 46 articles, 14 different measures were utilised to report model performance. An overview of performance measures reported per ML approach is given in Table 2. For classification models, accuracy, area under the receiver operating characteristic curve (AUC-ROC), sensitivity, and specificity were most frequently used across all ML approaches. All four registered regression models were realised by means of DNNs and evaluated by means of the correlation coefficient.

**Table 2:** Respective number of reviewed articles from 2022, in which a certain measure was used to report classification or regression performance of a certain machine learning approach. In case of more than one classification or regression task investigated within one and the same article, respective measures are counted only once. Both measures and approaches are listed (i) in decreasing order according to the number of articles (given in brackets), and (ii) alphabetically.

	Measure\Approach	RF (24)	DNN (23)	SVM (22)	LR (15)	DT (14)	KNN (6)	NN (6)	NB (4)	LDA (3)	DT+NN (1)	HMM (1)
Classification	Accuracy (31)	16	16	16	8	11	5	5	3	1	1	1
	AUC-ROC (28)	16	12	16	13	11	4	6	3	2	1	1
	Sensitivity (28)	14	12	14	8	10	3	5	3	3	1	
	Specificity (24)	12	11	12	7	9	3	5	1	3	1	
	PPV (16)	8	6	6	4	5	1	1	1			
	F1 score (13)	6	8	8	5	6	2	2	1	2		1
	NPV (7)	5	1	4	2	1						
	AUC-PR (6)	4	2	3	4	2	1	2		1	1	
	Brier score (2)	2		1	2	2						
	Kappa (2)	2		1								
	CC (1)		1	1	1	1				1		
	Error rate (1)	1	1	1	1	1		1				
Regr.	CC (4)		4									
	MAE (1)		1									
	STDAE (1)		1									

AUC-PR, area under the precision-recall curve; AUC-ROC, area under the receiver operating characteristic curve; CC, correlation coefficient; DNN, deep neural network; DT, decision tree; HMM, hidden Markov model; KNN, k-nearest neighbors; LDA, linear discriminant analysis; LR, logistic regression; MAE, mean absolute error; NB, naïve Bayes; NN, neural network; NPV, negative predictive value; PPV, positive predictive value; regr., regression; RF, random forest; STDAE, standard deviation of absolute error; SVM, support vector machine.

With regard to the performance itself, most studies demonstrated a basic feasibility of the investigated task with results fairly above chance level (e.g., 24, 31, 50, 52). In other studies (nearly) perfect performance was reached (26, 30, 34, 47–49, 53–57).

## 4 Discussion

In this work, we systematically reviewed articles on ML for detection and prediction of disease (status) in infants. Although it is still a relatively underrepresented application field of AI as compared to others, such as cancer, mental diseases, and chronic diseases (12–14, 58), our systematic review shows a clear increase of research activity from 2018 to 2022: More than twice as many articles were published in the year 2022 than in 2018. Interestingly, we revealed a slight stagnation of published articles in the year 2021. This might be due to the COVID-19 pandemic: The prevention of COVID-19 came along with remarkable challenges for the acquisition of research data and this was even more rigorous in vulnerable populations such as (preterm born) infants.

A wide variety of medical conditions covering twelve ICD-11 categories were investigated, reflecting the great potential of AI for infant healthcare. Some of these conditions mainly affect infants born preterm, such as bronchopulmonary dysplasia, necrotising enterocolitis and retinopathy of prematurity. A great proportion of the included articles focused on these conditions. Another major class of medical conditions subsume conditions that are usually recognised at a later time in development, such as autism spectrum disorder or deficits in various developmental domains. The studies on infants later diagnosed with a developmental disorder aimed to predict the condition based on clinical information gathered in infancy (e.g., 24, 50). Such a strategy could help to advance earlier diagnosis and intervention for affected children. The fact that we found partly overlapping authors for different articles on one and the same medical condition, such as retinopathy of prematurity, indicates that there seem to be specialised labs/research consortia actively driving ML research in specific diseases.

A multitude of different data types were used to automatically detect or predict a disease or disease status in infants. Almost 40% of the articles from 2022 relied on clinical and demographic information. The frequent use of this data type might be related to the facts that it subsumes diverse information about the participants, including partly even longitudinal data (e.g., 35, 36), and that it was often used in addition to other data types; Figure 2 shows that 18 articles used clinical and demographic information, but only 7 of them exclusively used this data for the detection or prediction of disease (status) in infants (Figure 2b). Some studies even relied on dozens or hundreds of features belonging to this data type fed into their ML algorithms. We are aware that the data type clinical and demographic information partly overlaps with the data type laboratory data. The main reason for that is that most articles did not describe how they collected certain clinical features. We still decided not to combine these two data types as we found it interesting to reveal how many studies mainly focused on the analysis of blood and stool samples for automatic detection and prediction of disease (status). However, we encourage future studies to describe their underlying data in more detail in order to get a more thorough insight in the worthiness of specific data for the detection and prediction of medical conditions in infants. Many reviewed studies used image-based data types, namely X-ray, MRI, photography, and video. The important role of image-based data in this field becomes especially obvious when considering the frequent exclusive use of these data for the detection or prediction of disease (status) in infants (Figure 2b). This is a similar picture as known from non-infant-centred application fields of AI in healthcare, such as from the detection of cancer (e.g., 59–61). In contrast to those widely used visual data, only a single of the included articles from 2022 relied on audio data (62). Also electrocardiography (ECG), electroencephalography (EEG), and thermal data are relatively underrepresented. In case of EEG, the fixation of electrodes on the infant’s scalp together with the potential use of conductive paste might cause discomfort. This can be an explanation for EEG currently not being among the top choices for that age. Nevertheless, studies based on the underrepresented data types revealed basic feasibility (i.e., results above chance level; 26, 50, 63) with regard to their respective tasks, just as studies exploiting the more frequently used data types did (28, 39, 55). This indicates not yet fully recognised potential of currently underrepresented data types for automatic disease (status) detection and prediction in infants. Future studies should consider these data types as an alternative or addition to clinical and demographic information, laboratory data, and visual data. As each data type comes along with specific advantages and disadvantages regarding reliability, acquisition costs, acquisition comfort for the patient, processing and storage requirements, etc., the combination of different data types could increase robustness. Nevertheless, the choice of data type ultimately depends on the investigated medical condition and its related symptoms. Furthermore, it might be most sustainable to exploit data types which are collected as part of a condition-related clinical protocol anyway.

With regard to applied ML approaches, our review clearly shows a trend towards DNNs. This is in line with recent developments in the general field of AI (64). Nevertheless, many studies still rely on traditional ML methodology, such as random forests or support vector machines, or provide results obtained via traditional approaches as baselines alongside DNN results. Despite rapid recent developments in the field of explainable artificial intelligence (XAI)—a branch of AI trying to make ML models better understandable to humans (65)—our finding might indicate that—even coming along with better performances—insufficient explainability of DNNs as compared to traditional ML approaches still represents an unsolved ethical issue, especially in healthcare-related application areas (66). Another reason might be that many studies using AI for a novel medically relevant task “just” aim to demonstrate basic feasibility—for which usually traditional ML is applied—rather than trying to develop a perfectly performing, ready-to-use AI system—which would most probably rely on a sophisticated DNN architecture and involve a number of additional ethical and political issues regarding liability, regulation of application, and costs (67). Besides, the question of using a traditional ML approach or a DNN approach is also a matter of available (training) data. Especially in niche fields of medical research—this includes AI for disease (status) detection or prediction in infants—datasets are often comparatively small. This makes traditional ML better suited than deep learning, which normally necessitates much more training data in order to effectively configure the complex underlying network architectures.

Finally, our review suggests that according to reported performances some investigated tasks could actually be resolved by or in assistance with AI in clinical settings in the near future. However, it has to be kept in mind that performance achieved in the framework of a research study certainly depends on several factors, such as the participant sample, the type, quality, quantity, and partitioning of available data, or the evaluation strategy. Moreover, our review shows that in highly interdisciplinary fields, such as automatic disease (status) detection and prediction in infants, a variety of different performance measures, such as accuracy, AUC-ROC, sensitivity, and specificity for classification model evaluations, and combinations of performance measures are reported. This can be explained by discipline-specific conventions as well as study design-, dataset- and application-specific advantages and disadvantages of each measure. For example, accuracy is very simple to compute, but as it “just” indicates the proportion of correct predictions among all predictions—irrespective of classes, it can be misleading in situations with class-imbalanced datasets. Moreover, accuracy does not directly take into account false negatives and false positives. In contrast, the AUC-ROC reflects the trade-off between the true positive rate (sensitivity, recall) and the false positive rate at different decision thresholds. Thereby, it indicates the model’s ability to distinguish between positive and negative cases. The curve itself is a useful instrument to select appropriate thresholds for the requirements of specific applications. Sensitivity is particularly useful in tasks with a high cost of false negatives, e.g., when trying to detect rare events, whereas specificity quantifies how well a model identifies (true) negatives. In medical research, sensitivity and specificity are usually reported together, which is also confirmed by our review study. In 24 out of 28 classification model evaluations, in which sensitivity was reported, specificity was reported as well. Thus, sensitivity was reported without specificity in four cases only. In contrast, specificity was never reported without sensitivity. All study-dependent aspects taken together make it hard to compare performances between different studies—even if the same tasks were investigated—or to assess, whether an approach is actually “ready” for real-world application.

### 4.1 Limitations

We decided to search the databases PubMed and IEEE Xplore. Most probably, an additional search in other databases would have revealed additional relevant articles. Although we carefully selected our search terms based on our experience, additional search terms such as names of specific ML algorithms going beyond umbrella terms, medical conditions listed in ICD-11, or symptoms might have brought further suitable matches including for example articles on Rett syndrome or fragile X syndrome focusing on infant vocalisation analysis (68), as well as articles on neonatal seizures (69) and cerebral palsy (70) focusing on electrophysiological analysis. However, a broad extension of search terms would have caused an exponential increase of first search hits, while the number of finally included articles might have just minimally grown. A more extended search would have also been beyond the scope of this work, as our aim was to give a representative overview, not to uncover every single detail. Next, we need to point out that the manuscript does not fully meet PRISMA criteria (71) in terms of risk of bias assessment, synthesis methods, and reporting of study selection processes. With regard to model performances, we decided not to discuss absolute values as a comparison across the different studies would be meaningless due to study-specific differences, e.g., in the investigated medical condition, data type, task, sample size, etc. However, for the interested reader, all reported performance values are given in Table S1 in the Supplementary Material. Finally, at a time when LLMs have become a major game changer in several areas, it might be a limitation of this work to have focused on the pre-LLM period only. However, in our opinion it is still too early to capture the whole impact of this new technology, especially in the healthcare domain. That is why we leave another review on the early LLM years for future work.

### 4.2 Conclusion

This work clearly shows that AI applications have been gaining momentum in the field of disease (status) detection and prediction in infants over the last couple of years before the release of ChatGPT; a further boost through LLMs is highly expected, given their broad factual and semantic medical knowledge as well as their capability of medical reasoning and processing complex concepts (72). We revealed that AI had already been used for a variety of medical conditions and diverse data types with overall promising performances and DNNs representing the most popular ML approach. Taken together, our results suggest that AI has a great potential to considerably improve diagnostic procedures for one of the most vulnerable population groups.

## Footnotes

^1^Please note that we use “detection” as a medical term throughout this work, even though presented studies actually deal with classification or regression tasks from a technical perspective.
