# Supplementary material for: Focused review on artificial intelligence for disease detection in infants
Source: Front Digit Health. 2024 Nov 25;6:1459640. doi: 10.3389/fdgth.2024.1459640 (PMC11625793; doi:10.3389/fdgth.2024.1459640)
Supplement: Supplementary file 1 [file Table1.pdf]

SUPPLEMENTARY MATERIAL

Table 1 Overview of articles published in 2022. Articles are listed (i) in decreasing order according to the number of articles assigned to the respective ICD-11 disease categories, (ii) alphabetically according to ICD-11 disease category/medical condition, and (iii) alphabetically according to (first) author's name. In the column 'Task(s)', we specify whether a given task is a detection (⇓) or prediction (⇒) task, and whether it focuses on the detection/prediction of a condition (●) or the condition status (◐). For each article, we present the best/most significant performance as reported by the authors and/or evident from the given results, i. e., best results across the reported performance measures. The respective best approach/task configuration is highlighted in bold font, if applicable. Decimal places for performance measures are given as reported in the original article, respectively. For further details, the reader is referred to the original articles.

ICD-11	Condition	Reference	Cohort	Data	Task(s)	Approach	Performance
CPP	Bronchopulmonary dysplasia	Leigh et al. (2022)	689 preterm infants born at GA≤30w3d	Perinatal features (e. g., ethnicity, birth weight, maternal smoking), respiratory support mode	⇒ ◐ With vs without bronchopulmonary dysplasia-free survival at 36w (2-class)	RF	Acc: 0.810<br>AUC-ROC: 0.899<br>NPV: 0.773<br>PPV: 0.855
CPP	Bronchopulmonary dysplasia	Patel et al. (2022)	38 preterm infants (12 females) born at GA≤30w; birth weight<1,250g	Demographic data (e. g., GA, gender), respiratory data (e. g., ventilator type, fraction of inspired oxygen)	⇒ ◐ With vs without respiratory support needs at **36w**, 37w, and 40w (2-class)	**RF**, SVM	Acc: 0.870<br>AUC-ROC: 0.934<br>F1: 0.857<br>κ: 0.739<br>NPV: 0.839<br>PPV: 0.913<br>Sens: 0.808<br>Spec: 0.929
CPP	Bronchopulmonary dysplasia	Sun et al. (2022)	403 premature infants born at GA≤32w: 128 with and 275 without bronchopulmonary dysplasia	Chest X-ray images, congenital attributes (e. g., GA, gender, Apgar score)	⇓ ● With vs without bronchopulmonary dysplasia (2-class)	**DNN-2+**, DT, HMM, SVM	Acc: 0.810<br>AUC-ROC: 0.863<br>F1: 0.800
CPP	Bronchopulmonary dysplasia	Xing et al. (2022)	121 premature infants with bronchopulmonary dysplasia; severity: 43 mild, 47 moderate, 31 severe	Chest X-ray images	⇒ ◐ Severity of bronchopulmonary dysplasia on 28th d of oxygen inhalation (3-class)	DNN-2+	Acc: 0.9558<br>F1: 0.9561<br>PPV: 0.9561<br>Sens: 0.9567<br>Spec: 0.9698

ICD-11	Condition	Reference	Sample	Data	Task(s)	Approach	Performance
CPP	Hypoxic-ischemic encephalopathy	Jeong et al. (2022)	15 term newborns with hypoxic-ischemic encephalopathy; outcome: 5 normal, 5 abnormal motor, 5 death	DWI tractography data (first four weeks of life)	⇒ ◐ Outcome at 2y (3-class)	RF	Acc: 0.987 Sens: 0.997 Spec: 1.000
CPP	Necrotising enterocolitis	Cho et al. (2022)	10,353 infants with VLBW (5,115 males), 704 of them with necrotising enterocolitis	Perinatal predictors (e. g., maternal age, cesarean section), environmental predictors (e. g., average ambient temperature for birth year)	⇓ ● With vs without necrotising enterocolitis (2-class)	DNN-2, DT, **LR**, NB, RF, SVM	Acc: 0.932 AUC-ROC: 0.730
CPP	Necrotising enterocolitis	Lin et al. (2022)	261 preterm infants, 75 of them with necrotising enterocolitis	Stool microbiota DNA sequences, clinical and demographic details (e. g., GA at birth, feeding mode)	⇓ ● With vs without necrotising enterocolitis (2-class)	**DNN-2+**, LR, SVM	AUC-PR: 0.749 AUC-ROC: 0.915
CPP	Necrotising enterocolitis	Lueschow et al. (2022)	219 infants; 102 with necrotising enterocolitis (57 males), 117 without necrotising enterocolitis (61 males)	67 features on systemic findings, abdominal exam findings, and radiographic characteristics (e. g., apnea, pneumatosis, occult rectal bleeding)	⇓ ● With vs without necrotising enterocolitis (2-class)	**DT**, KNN, NB, NN, RF, SVM	Acc: 0.800 AUC-ROC: 0.804 Sens: 0.826 Spec: $\approx$1
CPP	Necrotising enterocolitis	Qi et al. (2022)	45 newborns with necrotising enterocolitis: 37 preterm and 8 term infants	115 radiomic features (e. g., entropy, skewness), 79 clinical features (e. g., thrombocytosis, oxygen saturation)	⇒ ◐ Necrotising enterocolitis surgery need (2-class)	LR, **RF**, SVM	AUC-ROC: 0.80

ICD-11	Condition	Reference	Sample	Data	Task(s)	Approach	Performance
CPP	Neonatal opioid withdrawal syndrome	Manigault et al. (2022)	65 neonates (36 females) exposed or not exposed to opioids: 19 with and 46 without pharmacological treatment for neonatal opioid withdrawal syndrome	Audio recordings of newborn cries	⇒ ◐<br>Pharmacological treatment need for neonatal opioid withdrawal syndrome (2-class)	RF	Acc: 0.85<br>AUC-PR: 0.72<br>AUC-ROC: 0.90<br>κ (Cohen): 0.66<br>NPV: 0.95<br>PPV: 0.68<br>Sens: 0.89<br>Spec: 0.83
CPP	Parenteral nutrition-associated cholestasis	Moutinho et al. (2022)	60 preterm and term neonates who received parenteral nutrition: 19 (11 males) with and 41 (25 males) without parenteral nutrition-associated cholestasis	Stool metabolomics	⇓ ●<br>With vs without parenteral nutrition-associated cholestasis (2-class)	RF	Acc: 0.85
CPP	Postnatal intestinal perforation	Son et al. (2022)	12,555 VLBW infants: 521 with intestinal perforation associated with necrotising enterocolitis (285 males); 208 with spontaneous intestinal perforation (130 males), 11,826 controls (5,919 males); additional 57 VLBW infants for testing	Maternal and perinatal factors (e. g., gestational diabetes mellitus, Apgar score, birth head circumference)	① ⇓ ●<br>With vs without intestinal perforation associated with necrotising enterocolitis (2-class)<br>② ⇓ ◐<br>With vs without spontaneous intestinal perforation (2-class)	**DNN-2+**, DT, KNN, LR, RF, SVM	①<br>AUC-ROC: 1.0000<br>F1: 0.9076<br>②<br>AUC-ROC: 0.9364<br>F1: 0.7179
MBND	Autism spectrum disorder	Doi et al. (2022)	41 infants born at term (27 males); 7 of them at high risk for autism spectrum disorder; age: 4m	Video recordings of spontaneous movements	⇒ ●<br>High vs low risk for autism spectrum disorder at 18m (2-class)	LDA, LR, **NN**	AUC-PR: 0.688<br>AUC-ROC: 0.903<br>Sens: 0.714<br>Spec: 0.971<br>F1: 0.769

ICD-11	Condition	Reference	Sample	Data	Task(s)	Approach	Performance
MBND	Autism spectrum disorder	Tye et al. (2022)	216 infants: 68 (44% males) at low risk for autism spectrum disorder and 148 (49% males) at high risk for autism spectrum disorder, 33/148 (76% males) with and 115/148 (42% males) without autism spectrum disorder; age: 8m	EEG data	⇒ ●<br>With vs without autism spectrum disorder at 36m (2-class)	SVM	Acc: 0.757<br>AUC-ROC: 0.771<br>Sens: 0.735<br>Spec: 0.778<br>NPV: 0.746<br>PPV: 0.768
MBND	Cognitive deficits	Ali et al. (2022)	343 very preterm infants (174 males); of these 103 children (49 males) had a cognitive assessment at 2y corrected age: 24 infants (15 males) with and 79 infants (34 males) without cognitive deficits; mean age: 42.7w PMA	Functional connectome data	⇒ ●<br>High vs low risk for cognitive deficits at 2y (2-class)	**DNN-2+**, DT, KNN, SVM	Acc: 0.710<br>AUC-ROC: 0.750<br>Sens: 0.704<br>Spec: 0.715
MBND	Cognitive deficits	Chen et al. (2022a)	80 very preterm infants (41 males) born at GA≤31w; 31 with and 49 without cognitive deficits at 2y corrected age; mean age: 40.4w PMA	Structural connectome data	① ⇒ ●<br>High vs low risk for cognitive deficits at 2y (2-class)<br>② ⇒ ◐<br>Cognitive scores at 2y (regression)	DNN-2+	①<br>Acc: 0.816<br>AUC-ROC: 0.81<br>Sens: 0.783<br>Spec: 0.836<br>②<br>CC (Pearson): 0.41<br>MAE*: 14.2<br>STDAE*: 9.3<br>*scale range: 40–160

ICD-11	Condition	Reference	Sample	Data	Task(s)	Approach	Performance
MBND	Cognitive deficits	Li et al. (2022b)	276 very preterm infants from 2 cohorts: **207 (69 (43 males) with and 138 (67 males) without cognitive deficits at 2y corrected age**) + 69 (10 (5 males) with and 59 (34 males) without cognitive deficits at 2y corrected age); age: 39–44w PMA	Structural MRI data	⇒ ● High vs low risk for cognitive deficits at 2y (2-class)	DNN-2+, DT, **DT+NN (ensemble learning)**, KNN, LR, NN, RF, SVM	Acc: 0.713<br>AUC-PR: 0.56<br>AUC-ROC: 0.74<br>Sens: 0.706<br>Spec: 0.726
MBND	Developmental dyslexia	Yu et al. (2022)	98 infants (47 males), 35 (20 males) of them with and 63 (27 males) without familial history of dyslexia; mean age: 8.5m	Resting-state functional MRI data	⇓ ● With vs without familial history of dyslexia (2-class)	SVM	Acc: 0.55<br>Sens: 0.54<br>Spec: 0.56
MBND	Language deficits	Valavani et al. (2022)	89 preterm neonates born at GA≤33w, 14 (12 males) of them with and 75 (35 males) without language deficits at 2y corrected age; age: 38–42w GA	Diffusion MRI data; clinical perinatal factors	⇒ ● With vs without language deficits at 2y (2-class)	RF	Acc: 0.91<br>Sens: 0.86<br>Spec: 0.96
DA	Coarctation of aorta	Bahado-Singh et al. (2022)	24 newborns (16 males) with and 16 newborns (9 males) without coarctation of aorta	Blood samples	⇓ ● With vs without coarctation of aorta (2-class)	**DNN-2+**, LDA, RF, SVM	AUC-ROC: 0.97<br>Sens: 0.95<br>Spec: 0.98
DA	Congenital heart disease	Ruiz et al. (2022)	446 infants (258 males) with single ventricle or shunt-dependent congenital heart disease with or without deterioration events during stay at intensive care unit; age: <6m	Clinical data from electronic health records on vital signs, medications, laboratory tests, and diagnoses	⇒ ◐ With vs without clinical deterioration in 1, 2, **4**, 6, or 8 hours in congenital heart disease (2-class)	**DT**, LR, RF	AUC-ROC: 0.923<br>Brier score: 0.571<br>PPV: 0.776<br>Sens: 0.881<br>Spec: 0.862

ICD-11	Condition	Reference	Sample	Data	Task(s)	Approach	Performance
DA	Congenital heart disease	Shi et al. (2022)	536 infants (320 males) with congenital heart disease who underwent surgical repair at 4.56±3.12m; 97 of them had underweight 1y after surgery; additional 186 infants (108 males) with congenital heart disease for testing	Data from electronic medical records and self-administered questionnaires (e. g., treatments, biochemical tests, gestational vitamin, and mineral supplementation)	⇒ ◐ With vs without ① underweight, ② stunted, or ③ wasting status ***3m**, *6m, and **1y** after congenital heart disease surgery (2-class) *① only	**DT**, LR, NN, SVM	① Acc: 0.84 AUC-ROC: 0.91 Sens: 0.84 Spec: 0.93 ② AUC-ROC: 0.72 ③ AUC-ROC: 0.80
DA	Craniosynostosis	Mizutani et al. (2022)	56 individuals with non-syndromic craniosynostosis (age: 1–81m): 17 (15 males) with scaphocephaly, 28 (18 males) with trigonocephaly, 8 (4 males) with anterior plagiocephaly, 3 (1 male) with posterior plagiocephaly; 24 individuals (15 males) without craniosynostosis (age: 3–72m)	Skull X-ray images	⇓ ● ◐ With specific type of non-syndromic craniosynostosis (4 types) vs without non-syndromic craniosynostosis (5-class)	DNN-2+	Acc: 1 PPV: 1 Sens: 1 Spec: 1
DA	Craniosynostosis	Schaufelberger et al. (2022)	189 infants with craniosynostosis: 22 with coronal, 56 with metopic, 111 with sagittal synostosis; 178 infants without craniosynostosis	Representations of infants' head shapes created from 3D photogrammetric surface scans as 2D distance maps	⇓ ● ◐ With specific type of craniosynostosis (3 types) vs without craniosynostosis (4-class)	DNN-2+	Acc: 0.984
DA	Developmental dysplasia of hip	Fraiwan et al. (2022)	120 infants with and 234 infants without developmental dysplasia of hip; mean age: 4.5±0.83m	Pelvic X-ray images	⇓ ● With vs without developmental dysplasia of hip (2-class)	DNN-2+	Acc: 0.963 F1: 0.95 PPV: 0.906 Sens: 1 Spec: 0.943

ICD-11	Condition	Reference	Sample	Data	Task(s)	Approach	Performance
DNS	Cerebral palsy	Groos et al. (2022)	557 infants (310 males) with high risk of perinatal brain injury, 84 of them diagnosed with cerebral palsy at a mean age of 3.4y; age: 9–18w corrected age	Video recordings of spontaneous movements	⇒ ● With vs without cerebral palsy (2-class)	DNN-2+	Acc: 0.906<br>NPV: 0.949<br>PPV: 0.682<br>Sens: 0.714<br>Spec: 0.941
DNS	Cerebral palsy	Zhang et al. (2022)	Infants with or without cerebral palsy; age: **6m (dataset 1)**, 36–60w (dataset 2)	RGB-D video sequences of spontaneous movements	⇒ ● With vs without cerebral palsy (2-class)	**DNN-2+**, DT, LDA, LR, SVM	Acc: 1<br>F1: 1<br>CC (Matthew): 1<br>Sens: 1<br>Spec: 1
DNS	Neonatal intraventricular hemorrhage	Jin et al. (2022)	5,926 neonates with intraventricular hemorrhage (3,272 males); 67.6% with GA$<$32w, 81.2% with LBW	Maternal and neonatal characteristics (e. g., mother's age, neonatal risk factors, pregnancy risk factors)	⇒ ◐ Death within 1m of intraventricular hemorrhage diagnosis (2-class)	DT, LR, **RF**, SVM	AUC-PR: 0.581<br>AUC-ROC: 0.882<br>Brier score: 0.067<br>NPV: 0.917<br>PPV: 0.658<br>Sens: 0.279<br>Spec: 0.982
DNS	Neuromotor disorders	Moro et al. (2022)	142 preterm infants (52 males) born at 29$\pm$2w; 59 of them with neuromotor disorders; age: 40w GA	Video recordings of spontaneous movements	⇒ ● With vs without neuromotor disorders at 30m (2-class)	**DNN-2+**, RF, SVM	Acc: 0.857<br>Sens: 0.818<br>Spec: 0.823
DNS	WEST epilepsy syndrome	Chen et al. (2022b)	12 infants with WEST syndrome	ECG data	⇓ ◐ Seizure vs 0–10, 10–20, 20–30, or **more than 30 min** from seizure onset (2-class)	**DNN-2**, KNN, RF, SVM	Acc: 0.9558<br>F1: 0.9449<br>PPV: 0.9376
DVS	Retinopathy of prematurity	Campbell et al. (2022)	Infants from 2 separate datasets with classified mode and stage of retinopathy of prematurity	Retinal images	⇓ ◐ Severity of plus disease in retinopathy of prematurity (regression)	DNN-2+	CC (Pearson): 0.90

ICD-11	Condition	Reference	Sample	Data	Task(s)	Approach	Performance
DVS	Retinopathy of prematurity	Cole et al. (2022)	15 infants: 4 with plus disease, 9 with preplus disease, 2 without retinopathy of prematurity	Retinal images	⇓ ◐<br>Severity of plus disease in retinopathy of prematurity (regression)	DNN-2+	CC: 0.89
DVS	Retinopathy of prematurity	Li et al. (2022a)	2,801 infants with or without retinopathy of prematurity; birth weight<2,500g or GA<37w	Retinal images	① ⇓ ●<br>With vs without (**types of**) retinopathy of prematurity (2-class)<br>② ⇓ ◐<br>Severity of retinopathy of prematurity (regression)	DNN-2+	①<br>AUC-ROC: 0.986<br>②<br>CC (Spearman): 0.758
DVS	Retinopathy of prematurity	Lu et al. (2022)	Preterm infants from 7 institutions with plus, preplus, or no plus disease	Retinal images	⇓ ◐<br>With plus vs with preplus vs without plus disease in retinopathy of prematurity (3-class)	DNN-2+	AUC-ROC: 0.96
DVS	Retinopathy of prematurity	Yang et al. (2022)	57 infants with and 57 infants without retinopathy of prematurity until 40w PMA; born at GA 20–37w	Metabolites	⇓ ●<br>With vs without retinopathy of prematurity (2-class)	RF	AUC-ROC: 0.766<br>Sens: 0.719<br>Spec: 0.719
IPD	Sepsis	Honoré et al. (2022)	118 VLBW infants (52 males), 10 of them with sepsis	ECG, chest impedance, pulse oximetry, demographic factors (e. g., gender), body weight	⇓ ●<br>With vs without sepsis (2-class)	**DNN-2+**, LR, NN	Acc (balanced): 0.66<br>AUC-ROC: 0.81
IPD	Sepsis	Rassels and French (2022)	26 preterm infants with or without sepsis	Thermal recordings	⇓ ●<br>With vs without sepsis (2-class)	DT, KNN, LR, RF, **SVM**	Acc: 0.805

ICD-11	Condition	Reference	Sample	Data	Task(s)	Approach	Performance
IPD	Sepsis	Stocker et al. (2022)	1685 neonates (989 males) born at GA>34w with antibiotic therapy due to suspected early onset sepsis within the first 72h of life, 28 of them (15 males) with proven sepsis	Biomarkers (e. g., C-reactive protein), risk factors (e. g., positive maternal group B streptococci status), clinical signs (e. g., apnea)	$\Downarrow$ ● With vs without early onset sepsis (2-class)	RF	AUC-PR: 0.2842<br>AUC-ROC: 0.8341
IPD	Serious bacterial infection	Martin et al. (2022)	15,074 children (8,423 males); age: 3m–18y; admitted to paediatric intensive care unit and underwent an infectious work-up; 4,788 of them (2,607 males) received antibiotics (**AEP**), 10,286 (5,816 males) did not receive antibiotics (AUP) before admission; 2,325 of AEP (1,239 males) had serious bacterial infection, 2,356 of AUP (1,279 males) had serious bacterial infection	Vital signs, laboratory, and clinical electronic health record data (e. g., age at admission, presence of complex chronic condition)	$\Downarrow$ ● With vs without serious bacterial infection (2-class)	LR, **RF**, SVM	AUC-ROC: 0.80<br>NPV: 0.97
SSCNEC	Postnatal growth failure	Han et al. (2022)	7.954 preterm infants with VLBW; age: at birth, **7d**, 14d, at discharge	Clinical variables (e. g., birth weight, intraventricular hemorrhage, treatments for patent ductus arteriosus, sepsis)	$\Rightarrow$ ● With vs without postnatal growth failure (2-class)	DNN-2, DNN-2+, **DT**, LR, NN, RF, SVM	Acc: 0.68<br>AUC-ROC: 0.74<br>Error rate: 0.32<br>F1: 0.67<br>PPV: 0.62<br>Sens: 0.73<br>Spec: 0.63
SSCNEC	Postnatal growth failure	Yoon et al. (2022)	7,954 preterm infants with VLBW; age: at birth, 7d, 14d, **28d**	Prenatal and postnatal factors (e. g., maternal hypertension, duration of ventilation, sepsis)	$\Rightarrow$ ● With vs without postnatal growth failure (2-class)	DT	Acc: 0.79<br>AUC-ROC: 0.88<br>F1: 0.70<br>PPV: 0.66<br>Sens: 0.75<br>Spec: 0.81

ICD-11	Condition	Reference	Sample	Data	Task(s)	Approach	Performance
DEM	Hearing loss	Venkataramana and Thilagam (2022)	Children with or without hearing loss; age: 0–4y	Prenatal, natal, and postnatal factors (e. g., history of hearing loss in family, delivery mode) and hearing test data (e. g., ear-wise tympanometry or reflexometry values)	$\Downarrow$ ● ◐ Level of hearing loss (49-class)	DT, NB, RF, **SVM**	Acc: 1<br>F1: 1<br>PPV: 1<br>Sens: 1
DRS	Neonatal chronic lung disease	Maeda et al. (2022)	30 preterm infants with chronic lung disease: 11 with mild, 19 with severe disease; age: 7d	Chest X-ray images	$\Downarrow$ ◐ Severity of chronic lung disease (2-class)	DNN-2+	Acc: 0.667<br>F1: 0.750<br>PPV: 0.714<br>Sens: 0.789
DS	Atopic dermatitis	Jiang et al. (2022)	88 children (56 males) with and 73 (33 males) without atopic dermatitis; age: 6–72m	Transcriptome and microbiota data	$\Downarrow$ ● With vs without atopic dermatitis (2-class)	LR, RF, **SVM**	Acc: 0.7333<br>AUC-ROC: 0.75<br>F1: 0.7778<br>PPV: 0.7000<br>Sens: 0.8750
ENMD	Diabetes mellitus type 1	Webb-Robertson et al. (2022)	655 children: 73 (33 males) with and 582 (319 males) without diabetes mellitus type 1 at 6y; age at inclusion: <4.5m	Genetic, immunologic, and metabolic features (e. g., human leukocyte antigen genotypes, piperidone, cystine)	$\Rightarrow$ ● With vs without diabetes mellitus type 1 at 6y (2-class)	NB	AUC-ROC: 0.84<br>Sens: 0.40
SWD	Sleep apnea	Bennet et al. (2022)	Infants with or without sleep apnea	ECG data	$\Downarrow$ ● With vs without sleep apnea (2-class)	RF	Acc: 0.8658

Abbreviations: Acc = accuracy; AUC-PR = area under the precision-recall curve; AUC-ROC = area under the receiver operating characteristic curve; CC = correlation coefficient; CPP = certain conditions originating in the perinatal period; d = day(s); DA = developmental anomalies; DEM = diseases of the ear or mastoid process; DS = diseases of the skin; DT = decision tree; DNN-2 = deep neural network with two hidden layers; DNN-2+ = deep neural network with more than two hidden layers; DNA = deoxyribonucleic acid; DNS = diseases of the nervous system; DRS = diseases of the respiratory system; DVS = diseases of the visual system; DWI = diffusion-weighted imaging; ECG = electrocardiogram; EEG = electroencephalography; ENMD = endocrine, nutritional or metabolic diseases; GA = gestational age; IPD = certain infectious or parasitic diseases; KNN = k-nearest neighbors; (V)LBW = (very) low birth weight; LDA = linear discriminant analysis; LR = logistic regression; m = month(s); MAE = mean absolute error; MBND = mental, behavioural or neurodevelopmental disorders; MRI = magnetic resonance imaging; NB = naïve Bayes; NN = neural network (with one hidden layer); NPV = negative predictive value; PMA = postmenstrual age; PPV = positive predictive value; RF = random forest; sens = sensitivity; spec = specificity; SSCNEC = symptoms, signs or clinical findings, not elsewhere classified; STDAE = standard deviation of absolute error; SVM = support vector machine; SWD = sleep-wake disorders; w = week(s); y = year(s).

REFERENCES

Ali, R., Li, H., Dillman, J. R., Altaye, M., Wang, H., Parikh, N. A., et al. (2022). A self-training deep neural network for early prediction of cognitive deficits in very preterm infants using brain functional connectome data. *Pediatric Radiology* 52, 2227–2240

Bahado-Singh, R. O., Vishweswaraiah, S., Aydas, B., Yilmaz, A., Saiyed, N. M., Mishra, N. K., et al. (2022). Precision cardiovascular medicine: Artificial intelligence and epigenetics for the pathogenesis and prediction of coarctation in neonates. *The Journal of Maternal-Fetal & Neonatal Medicine* 35, 457–464

Bennet, M. A., Subha, K., Kumutha, R., and Rajmohan, V. (2022). An effective method for distinguishing breathing and infant sleep apnea detection and prevention using Python. In *2022 International Conference on Computer, Power and Communications (ICCPC)* (IEEE), 433–438

Campbell, J. P., Chiang, M. F., Chen, J. S., Moshfeghi, D. M., Nudleman, E., Ruambivoonsuk, P., et al. (2022). Artificial intelligence for retinopathy of prematurity: Validation of a vascular severity scale against international expert diagnosis. *Ophthalmology* 129, e69–e76

Chen, M., Li, H., Fan, H., Dillman, J. R., Wang, H., Altaye, M., et al. (2022a). ConCeptCNN: A novel multi-filter convolutional neural network for the prediction of neurodevelopmental disorders using brain connectome. *Medical Physics* 49, 3171–3184

Chen, S., Zheng, R., Wang, T., Jiang, T., Gao, F., Wang, D., et al. (2022b). Deterministic learning-based WEST syndrome analysis and seizure detection on ECG. *IEEE Transactions on Circuits and Systems II: Express Briefs* 69, 4603–4607

Cho, H., Lee, E. H., Lee, K.-S., and Heo, J. S. (2022). Machine learning-based risk factor analysis of necrotizing enterocolitis in very low birth weight infants. *Scientific Reports* 12, 21407

Cole, E. D., Park, S. H., Kim, S. J., Kang, K. B., Valikodath, N. G., Al-Khaled, T., et al. (2022). Variability in plus disease diagnosis using single and serial images. *Ophthalmology Retina* 6, 1122–1129

Doi, H., Iijima, N., Furui, A., Soh, Z., Yonei, R., Shinohara, K., et al. (2022). Prediction of autistic tendencies at 18 months of age via markerless video analysis of spontaneous body movements in 4-month-old infants. *Scientific Reports* 12, 18045

Fraiwan, M., Al-Kofahi, N., Ibnian, A., and Hanatleh, O. (2022). Detection of developmental dysplasia of the hip in X-ray images using deep transfer learning. *BMC Medical Informatics and Decision Making* 22, 1–11

Groos, D., Adde, L., Aubert, S., Boswell, L., De Regnier, R.-A., Fjørtoft, T., et al. (2022). Development and validation of a deep learning method to predict cerebral palsy from spontaneous movements in infants at high risk. *JAMA Network Open* 5, e2221325

Han, J. H., Yoon, S. J., Lee, H. S., Park, G., Lim, J., Shin, J. E., et al. (2022). Application of machine learning approaches to predict postnatal growth failure in very low birth weight infants. *Yonsei Medical Journal* 63, 640

Honoré, A., Siren, H., Vinuesa, R., Chatterjee, S., and Herlenius, E. (2022). An LSTM-based recurrent neural network for neonatal sepsis detection in preterm infants. In *2022 IEEE Signal Processing in Medicine and Biology Symposium (SPMB)* (IEEE), 1–6

Jeong, J.-W., Lee, M.-H., Fernandes, N., Deol, S., Mody, S., Arslanturk, S., et al. (2022). Neonatal encephalopathy prediction of poor outcome with diffusion-weighted imaging connectome and fixel-based analysis. *Pediatric Research* 91, 1505–1515

Jiang, Z., Li, J., Kong, N., Kim, J.-H., Kim, B.-S., Lee, M.-J., et al. (2022). Accurate diagnosis of atopic dermatitis by combining transcriptome and microbiota data with supervised machine learning. *Scientific Reports* 12, 290

Jin, M. C., Parker, J. J., Rodrigues, A. J., Colón, G. D. R., Garcia, C. A., Mahaney, K. B., et al. (2022). Development of an integrated risk scale for prediction of shunt placement after neonatal intraventricular hemorrhage. *Journal of Neurosurgery: Pediatrics* 29, 444–453

Leigh, R. M., Pham, A., Rao, S. S., Vora, F. M., Hou, G., Kent, C., et al. (2022). Machine learning for prediction of bronchopulmonary dysplasia-free survival among very preterm infants. *BMC Pediatrics* 22, 1–12

Li, J., Huang, K., Ju, R., Chen, Y., Li, M., Yang, S., et al. (2022a). Evaluation of artificial intelligence–based quantitative analysis to identify clinically significant severe retinopathy of prematurity. *Retina* 42, 195–203

Li, Z., Li, H., Braimah, A., Dillman, J. R., Parikh, N. A., and He, L. (2022b). A novel ontology-guided attribute partitioning ensemble learning model for early prediction of cognitive deficits using quantitative structural MRI in very preterm infants. *NeuroImage* 260, 119484

Lin, Y. C., Salleb-Aouissi, A., and Hooven, T. A. (2022). Interpretable prediction of necrotizing enterocolitis from machine learning analysis of premature infant stool microbiota. *BMC Bioinformatics* 23, 1–29

Lu, C., Hanif, A., Singh, P., Chang, K., Coyner, A. S., Brown, J. M., et al. (2022). Federated learning for multicenter collaboration in ophthalmology: Improving classification performance in retinopathy of prematurity. *Ophthalmology Retina* 6, 657–663

Lueschow, S. R., Boly, T. J., Jasper, E., Patel, R. M., and McElroy, S. J. (2022). A critical evaluation of current definitions of necrotizing enterocolitis. *Pediatric Research* 91, 590–597

Maeda, R., Fujita, D., Tanaka, K., Ozawa, J., Haga, M., Miyahara, H., et al. (2022). Predicting the severity of neonatal chronic lung disease from chest X-ray images using deep learning. In *2022 IEEE International Conference on Systems, Man, and Cybernetics (SMC)* (IEEE), 1543–1547

Manigault, A. W., Sheinkopf, S. J., Silverman, H. F., and Lester, B. M. (2022). Newborn cry acoustics in the assessment of neonatal opioid withdrawal syndrome using machine learning. *JAMA Network Open* 5, e2238783

Martin, B., DeWitt, P. E., Scott, H. F., Parker, S., and Bennett, T. D. (2022). Machine learning approach to predicting absence of serious bacterial infection at PICU admission. *Hospital Pediatrics* 12, 590–603

Mizutani, K., Miwa, T., Sakamoto, Y., and Toda, M. (2022). Application of deep learning techniques for automated diagnosis of non-syndromic craniosynostosis using skull. *Journal of Craniofacial Surgery* 33, 1843–1846

Moro, M., Pastore, V. P., Tacchino, C., Durand, P., Blanchi, I., Moretti, P., et al. (2022). A markerless pipeline to analyze spontaneous movements of preterm infants. *Computer Methods and Programs in Biomedicine* 226, 107119

Moutinho, T. J., Powers, D. A., Hanson, G. F., Levy, S., Baveja, R., Hefner, I., et al. (2022). Fecal sphingolipids predict parenteral nutrition-associated cholestasis in the neonatal intensive care unit. *Journal of Parenteral and Enteral Nutrition* 46, 1903–1913

Patel, M., Sandhu, J., and Chou, F.-S. (2022). Developing a machine learning-based tool to extend the usability of the NICHD BPD outcome estimator to the Asian population. *Plos One* 17, e0272709

Qi, G., Huang, S., Lai, D., Li, J., Zhao, Y., Shen, C., et al. (2022). An improved joint non-negative matrix factorization for identifying surgical treatment timing of neonatal necrotizing enterocolitis. *Bosnian Journal of Basic Medical Sciences* 22, 972

Rassels, K. and French, P. (2022). Bio-remote sensing in predicting infection in neonates with thermal imaging and machine learning. In *2022 Smart Systems Integration (SSI)* (IEEE), 1–4

Ruiz, V. M., Goldsmith, M. P., Shi, L., Simpao, A. F., Gálvez, J. A., Naim, M. Y., et al. (2022). Early prediction of clinical deterioration using data-driven machine-learning modeling of electronic health records. *The Journal of Thoracic and Cardiovascular Surgery* 164, 211–222

Schaufelberger, M., Kühle, R. P., Kaiser, C., Wachter, A., Weichel, F., Hagen, N., et al. (2022). CNN-based classification of craniosynostosis using 2D distance maps. In *2022 44th Annual International Conference of the IEEE Engineering in Medicine & Biology Society (EMBC)* (IEEE), 446–449

Shi, H., Yang, D., Tang, K., Hu, C., Li, L., Zhang, L., et al. (2022). Explainable machine learning model for predicting the occurrence of postoperative malnutrition in children with congenital heart disease. *Clinical Nutrition* 41, 202–210

Son, J., Kim, D., Na, J. Y., Jung, D., Ahn, J.-H., Kim, T. H., et al. (2022). Development of artificial neural networks for early prediction of intestinal perforation in preterm infants. *Scientific Reports* 12, 12112

Stocker, M., Daunhawer, I., Van Herk, W., El Helou, S., Dutta, S., Schuerman, F. A., et al. (2022). Machine learning used to compare the diagnostic accuracy of risk factors, clinical signs and biomarkers and to develop a new prediction model for neonatal early-onset sepsis. *The Pediatric Infectious Disease Journal* 41, 248–254

Sun, S., Ji, W., Tang, L., Zhang, X., Zhang, Y., and Feng, R. (2022). An interpretable causal approach for bronchopulmonary dysplasia prediction. In *2022 IEEE International Conference on Bioinformatics and Biomedicine (BIBM)* (IEEE), 1193–1200

Tye, C., Bussu, G., Gliga, T., Elsabbagh, M., Pasco, G., Johnsen, K., et al. (2022). Understanding the nature of face processing in early autism: A prospective study. *Journal of Psychopathology and Clinical Science* 131, 542

Valavani, E., Blesa, M., Galdi, P., Sullivan, G., Dean, B., Cruickshank, H., et al. (2022). Language function following preterm birth: Prediction using machine learning. *Pediatric Research* 92, 480–489

Venkataramana, P. K. and Thilagam, P. S. (2022). Hearing loss prediction in newborns, infants and toddlers using machine learning. In *2022 IEEE North Karnataka Subsection Flagship International Conference (NKCon)* (IEEE), 1–7

Webb-Robertson, B.-J. M., Nakayasu, E. S., Frohnert, B. I., Bramer, L. M., Akers, S. M., Norris, J. M., et al. (2022). Integration of infant metabolite, genetic, and islet autoimmunity signatures to predict type 1 diabetes by age 6 years. *The Journal of Clinical Endocrinology & Metabolism* 107, 2329–2338

Xing, W., He, W., Li, X., Chen, J., Cao, Y., Zhou, W., et al. (2022). Early severity prediction of BPD for premature infants from chest X-ray images using deep learning: A study at the 28th day of oxygen inhalation. *Computer Methods and Programs in Biomedicine* 221, 106869

Yang, Y., Yang, Q., Luo, S., Zhang, Y., Lian, C., He, H., et al. (2022). Comparative analysis reveals novel changes in plasma metabolites and metabolomic networks of infants with retinopathy of prematurity. *Investigative Ophthalmology & Visual Science* 63, 28–28

Yoon, S., Lee, S. M., and Kwon, Y. (2022). Prediction of postnatal growth failure in very low birth weight infants using artificial intelligence mode. In *2022 IEEE International Conference on Big Data (Big Data)* (IEEE), 6841–6843

Yu, X., Ferradal, S., Dunstan, J., Carruthers, C., Sanfilippo, J., Zuk, J., et al. (2022). Patterns of neural functional connectivity in infants at familial risk of developmental dyslexia. *JAMA Network Open* 5, e2236102

Zhang, H., Shum, H. P., and Ho, E. S. (2022). Cerebral palsy prediction with frequency attention informed graph convolutional networks. In *2022 44th Annual International Conference of the IEEE Engineering in Medicine & Biology Society (EMBC)* (IEEE), 1619–1625
